# The Association between Autoantibodies and Peripheral Neuropathy in Lupus Nephritis

**DOI:** 10.1155/2014/524940

**Published:** 2014-04-24

**Authors:** Yu-Jih Su, Chi-Ren Huang, Wen-Neng Chang, Nai-Wen Tsai, Chia-Te Kung, Wei-Che Lin, Chih-Cheng Huang, Chih-Min Su, Ben-Chung Cheng, Ya-Ting Chang, Cheng-Hsien Lu

**Affiliations:** ^1^Department of Internal Medicine, Kaohsiung Chang Gung Memorial Hospital and Chang Gung University College of Medicine, Kaohsiung 833, Taiwan; ^2^Department of Biological Science, National Sun Yat-sen University, Kaohsiung 80424, Taiwan; ^3^Department of Neurology, Kaohsiung Chang Gung Memorial Hospital and Chang Gung University College of Medicine, Kaohsiung 833, Taiwan; ^4^Department of Emergency Medicine, Kaohsiung Chang Gung Memorial Hospital and Chang Gung University College of Medicine, Kaohsiung 833, Taiwan; ^5^Department of Radiology, Kaohsiung Chang Gung Memorial Hospital and Chang Gung University College of Medicine, Kaohsiung 833, Taiwan

## Abstract

*Background and Aim*. The sensitivity and specificity of biomarkers used for predicting peripheral neuropathy in patients with systemic lupus erythematosus (SLE) and nephritis (SLE-LN) remain unsatisfactory. This study aimed to determine the autoantibodies levels in SLE-LN patients with peripheral neuropathy. *Methods*. Data of 559 SLE-LN patients were collected retrospectively, including titers of autoantibodies, electrodiagnostic studies, and clinical manifestations. *Results*. The neurologic manifestations of the SLE-LN patients were diverse and nonspecific. The prevalence rate of peripheral polyneuropathy was 2.68%, of which about 73.33% was mixed sensory-motor polyneuropathy. Numbness and functional gastrointestinal problems were the most prevalent symptoms and these were noted in every subtype of peripheral neuropathy. Among all the serology markers, anti-Ro was significantly associated with neuropathy related to SLE (*P* = 0.009). *Conclusion*. Peripheral neuropathy among LN patients is rare and may be easily overlooked. This study demonstrated that positive anti-Ro antibody may imply neuropathy in LN patients. Thus, anti-Ro can be considered a biomarker that should be added to the panel of conventional autoantibodies in LN patients.

## 1. Background

Peripheral neuropathy is a well-documented clinical manifestation of systemic lupus erythematosus (SLE) [1, 2], with a prevalence rate ranging from 2% to 27.8% [2, 3]. Several lines of evidence link the risk of neuropathy with the anti-phospholipid antibody and rheumatoid factor [4–6], as well as neuropsychiatric lupus with anti-Ro and anti-phospholipid antibody [7]. Some evidence links anti-ganglioside antibodies with neuropathy [8, 9], but other studies do not [10]. Peripheral neuropathy may be slowly progressive [11] or acutely devastating [12]. One longitudinal study shows no other factor associating neuropathy with lupus [12], but another reports that acute peripheral neuropathy may be associated with lupus nephritis under ciprofloxacin treatment [13].

In clinical practice, there are syndromes that overlap with lupus, including rheumatoid arthritis or vasculitis syndrome. Lupus nephritis (LN), a more definite and specific subgroup of lupus, is a major cause of morbidity and mortality in SLE and can affect up to 60% of SLE patients [14]. The five-year survival rate of LN has increased from 44% to 82% in the past 50 years. Despite improvements in medical treatment, LN affects 49–92% of SLE patients [14]. Furthermore, the presence of peripheral neuropathy in LN patients may be relevant for improving their lives, especially in terms of both quality of life and daily activities, including occupation and social interactions. Such complex situation poses a therapeutic challenge.

To date, very few researches have focused specifically on LN patients complicated by peripheral neuropathy [15–18]. Because of the possible benefits of therapeutic intervention, early diagnosis and treatment with better delineation of potential risk factors and clinical features are essential for this specific patient group.

## 2. Material and Methods

### 2.1. Study Design

Using preexisting standardized evaluation forms, the medical records of patients diagnosed with SLE and evidence of LN at Kaohsiung Chang Gung Memorial Hospital, a 2482-bed acute-care teaching hospital providing primary and tertiary referral care, were reviewed. Among the SLE-LN patients, electrodiagnostic tests were collected and evaluated to define the subtype of peripheral neuropathy after excluding patients according to the exclusion criteria. The demographic data, clinical presentation, and treatment were also collected.

### 2.2. Inclusion and Exclusion Criteria

The patient database in the study institution was searched for the ICD-9-CM code of SLE (710.0) and confirmed by clinical manifestations for the period of June 1, 2005, to June 1, 2011. Five hundred and eighty-two SLE patients with LN were collected. The 1997 update of the 1982 revised criteria was used for the diagnosis of SLE, while LN was diagnosed according to guidelines [19]. Patients with peripheral neuropathy related to end-stage renal disease, diabetes mellitus, and malignancy were excluded. In this study, patients fulfilled the diagnostic criteria of both electrophysiological study and typical clinical manifestations of peripheral neuropathy. Neurologists integrated the neurologic manifestations and findings of the nerve conduction study. The hospital's Institutional Review Committee on Human Research approved the study.

### 2.3. Electrodiagnostic Testing

The NCS/EMG examinations were done by the hospital's standard laboratory methods according to the recommended protocol of the American Association of Electrodiagnostic Medicine (AAEM) using a Nicolet Viking Select system (Nicolet Biomedical Inc., Madison, USA). All tests were done under similar temperature conditions in the same room. Skin temperature was maintained at ≥32°C.

### 2.4. Biochemical Analysis

The serum markers of autoantibodies were collected thoroughly from each patient, including anti-beta 2-glycoprotein I antibodies (a*β*2GP I), anti-cardiolipin IgM (aCL-IgM), anti-cardiolipin IgG (aCL-IgG), anti-Ro, anti-La, anti-RNP, anti-Jo-1, anti-Sm, anti-scl-70, anti-centromere (anti-cm), peri-nuclear anti-neutrophil cytoplasmic antibody (p-ANCA), and cytoplasmic anti-neutrophil cytoplasmic antibody (c-ANCA). All of the laboratory data were present with absolute values and all of the tests were done by the Thermo Scientific Phadia system by qualified technicians. Major symptoms that might be related to neuropathy were also collected from the charts.

### 2.5. Statistical Analysis

Data were expressed as mean ± SD or median (interquartile range). Categorical variables were compared using the Chi-square test or Fisher's exact test, as appropriate. The autoantibody levels were compared between the two groups. Univariate analyses were compared using the Mann-Whitney *U* test. All of the statistical analyses were conducted using the SAS software package, version 9.1 (2002, SAS Statistical Institute, Cary, North Carolina).

## 3. Result

### 3.1. Demographic Data

There were 1262 SLE patients treated in the hospital between June 1, 2005, and June 1, 2011, accounting for 3555 admissions. There were 582 SLE patients (46.04%) with kidney involvement and 2215 admissions (61.31% of all SLE admissions). Each patient had at least one admission. Among the 582 SLE-LN patients, three patients with diabetes and 20 with end-stage renal disease (ESRD) were excluded. Of the 559 patients finally enrolled in this study, 23 had a clinical diagnosis of peripheral neuropathy confirmed by electrophysiologic examinations. Among these 23 neuropathy patients, four patients with radiculopathy and four patients with median nerve neuropathy at wrist were further excluded. Thus, only 15 patients (2.68%) were diagnosed as definite neuropathy among the total 559 patients (Table 1).

### 3.2. Clinical, Serologic, and Laboratory Data

By subgroups of peripheral neuropathy, four had autonomic neuropathy, two had pure sensory neuropathy, and 11 had mixed sensory-motor polyneuropathy. Of the 11 with mixed sensory-motor polyneuropathy, two were also complicated with autonomic neuropathy.

The levels of autoantibodies were shown in Table 1. The clinical features and laboratory data of patients with and without neuropathy were compared. After Mann-Whitney *U* test of all aforementioned variables, only anti-Ro (*P* = 0.009) was independently associated with peripheral neuropathy among the SLE-LN patients.

The clinical symptoms of the 15 neuropathy patients (Table 2) were classified by clinical symptoms into eight categories: burning pain, sharp pain, extreme sensitivity to touch (allodynia), numbness, muscle weakness, bladder problem, abnormal blood pressure and heart rate, and functional gastrointestinal problems. Eleven patients had more than one category of symptoms. Numbness and functional gastrointestinal problems were the two most common symptoms (both *n* = 8). No patient presented with abnormal blood pressure and heart rate.

## 4. Discussion

Comorbid neuropathy and LN have been presented before but mostly as case reports before. Bódi et al. described an old lady who presented with progressive sensory-motor polyneuropathy and proteinuria. Her SLE was confirmed by biopsy and serology [17]. Acute inflammatory demyelinating polyneuropathy, Guillain-Barre syndrome, was described in several SLE patients with LN. Their treatments varied from cytotoxic agents to biologicals, which represented their complexity and severity [15, 16, 20, 21]. This study provides insights into the identification of neuropathy among LN patients based on clinical laboratory data and manifestations.

Differences in the relative prevalence of peripheral neuropathy in SLE vary with case determination, inclusion and exclusion criteria, and length of follow-up. The prevalence rate of peripheral neuropathy in SLE is a little bit lower than that of Sjögren's syndrome, which is characterized by positive anti-Ro autoantibodies. The prevalence rate of Sjögren's syndrome is between 8% and 62% [22]. In the present cohort, the prevalence rate is around 4.11%, which indicates that lupus nephritis may not be a predisposing factor of neuropathy. Current treatment strategies of lupus nephritis may not influence peripheral neuropathy. Interestingly, anti-Ro is associated with peripheral neuropathy among SLE-LN patients. This may explain the role of anti-Ro in the positive correlation of anti-Ro with neuropsychiatric SLE [7]. Moreover, the association between anti-Ro and polyneuropathy is also valid in all SLE patients in the present study (*P* = 0.0002, data not shown).

All of the different subtypes of peripheral neuropathy occur in SLE-LN patients. The most predominant is mixed sensory-motor polyneuropathy, which is seen in 11 of the 15 patients here (73.33%). The pathogenesis of median nerve neuropathy is either entrapment neuropathy [23] or polyneuropathy with or without focal neuropathy [24], and both have been excluded in this current study. Ultrasonography is a tool that can distinguish between these two different pathogeneses in the future [25].

The symptoms of polyneuropathy are nonspecific and diverse in this study cohort. Numbness and functional gastrointestinal problems are the most common presenting symptoms. Functional gastrointestinal problems and numbness can happen in any type of peripheral neuropathy, even in median nerve neuropathy and spinal radiculopathy. The combination of numbness on four extremities and functional gastrointestinal problems in an SLE-LN patient raise the index of suspicion on polyneuropathy in the future.

Anti-Ro autoantibody occurs in as high as one-third of SLE patients [26, 27] and is associated with Sicca syndrome and ocular damage [28] but is inversely associated with nephritis in one cohort study [29]. In this study, the prevalence rate of positive anti-Ro (cut-off value: 10 U/mL) is around 26.3% in all 559 SLE-LN patients. As such, positive anti-Ro antibody may be used as a complementary tool for diagnosing peripheral neuropathy.

This study has several limitations. First, this is a retrospective analysis and is therefore subject to bias of unmeasured factors. Second, peripheral neuropathy can be slowly progressive or acutely devastating, subtle, or even asymptomatic. In clinical practices, only LN patients with clinical peripheral neuropathy suspect or focal neurological signs receive electrodiagnostic studies. Third, LN patients with peripheral neuropathy and underlying conditions like ESRD, diabetes mellitus, and malignancy have been excluded. The findings may underestimate the “true” frequency of peripheral neuropathy in LN patients. Fourth, although the titers of autoantibodies in are semiquantitative, the level has a linear relationship with severity. However, the relationship between the severity of peripheral neuropathy and levels of anti-Ro antibody cannot be exactly evaluated in this study. Lastly, the choice of immunosuppressant therapeutic strategy for LN patients (e.g., drugs choice, dosage, and duration) may be different for each patient based on the preference of the attending physician. This may cause potential bias in the statistical analysis.

In conclusion, the occurrence of peripheral neuropathy among LN patients is rare and may be easily overlooked. This study demonstrates that positive anti-Ro antibody may imply neuropathy in LN patients and it can be considered a biomarker that should be added to the panel of conventional autoantibodies in LN.

## Figures and Tables

**Table 1:** Clinical and laboratory data of lupus nephritis with or without peripheral neuropathy.

	SLE-LN with neuropathy (*n* = 15)	SLE-LN without neuropathy (*n* = 544)	*P* value
Age	48.93 ± 15.02	41.5 ± 16.5	0.073
Male/female	0/15	16/528	ND
Classification of peripheral neuropathy^§^			
Autonomic neuropathy	4	0	ND
Mixed sensory-motor polyneuropathy	13	0	ND
Sensory polyneuropathy	2	0	ND
Autoantibody titers, median (IQR)			
Anti-*β*2 glycoprotein I	5.7 (2.65–6.25)	3.8 (2.4–5.6)	0382
Anti-cardiolipin IgG	21.6 (7.68–26.7)	6.5 (3.3–15.0)	0.071
Anti-cardiolipin IgM	3.8 (0.75–49.3)	2.2 (1.2–4.5)	0.429
Anti-Ro	240 (106–240.0)	92.3 (0.9–240.0)	0.009*
Anti-La	1.9 (1–51.4)	1.1 (0.4–6.3)	0.095
Anti-RNP	26.7 (2.3–77.9)	2.5 (1.4–27.9)	0.078
anti-Smith IgG	1.4 (0.5–2.9)	0.6 (0.3–2.7)	0.15
Anti-scl-70	0.35 (0.18–0.63)	0.4 (0.2–0.6)	0.699
Anti-Jo1	0.5 (0.1–0.5)	0.2 (0.1–0.5)	0.459
Anti-centromere IgG	0.5 (0.2–1.0)	0.5 (0.2–1.0)	0.457
p-ANCA	0.9 (0.6–2.1)	0.9 (0.5–1.4)	0.804
c-ANCA	1.1 (0.48–1.43)	0.9 (0.5–1.4)	0.855

^§^Four patients had both polyneuropathy and autonomic neuropathy.

SLE-LN: systemic lupus erythematosus with lupus nephritis; p-ANCA: peri-nuclear anti-neutrophil cytoplasmic antibody; c-ANCA: cytoplasmic anti-neutrophil cytoplasmic antibody; IQR: interquartile range; CI: confidence interval; ND: not done.

*P < 0.05.

**Table 2:** Symptoms of neuropathy among SLE-LN patients with peripheral neuropathy and without diabetes.

Symptoms of neuropathy (*n* = 15)	Autonomic neuropathy (*n* = 4)^*δ*^	Mixed sensory-motor polyneuropathy (*n* = 11)^*γ*^	Sensory polyneuropathy (*n* = 2)^†^
Burning sensation (*n* = 3)	0	2	1
Sharp and electric pain (*n* = 2)	0	2	0
Extreme sensitivity to touch (*n* = 2)	1	1	0
Numbness on four extremities (*n* = 8)	1	6	1
Muscle weakness or paralysis (*n* = 6)	1	5	0
Bladder symptoms (*n* = 1)	0	1	0
Functional GI problems (*n* = 8)	2	5	1

GI: gastrointestinal.

Note: functional GI problems include gastroesophageal reflux disease, irritable bowel syndrome, and idiopathic chronic constipation.

^*δ*^Two patients had the symptoms of numbness and functional GI problems.

^*γ*^One patient had overlapping bladder problem and numbness, three patients had overlapping functional GI problems and numbness, one patient had overlapping burning pain and electric-like pain, one patient had overlapping numbness and extreme sensitivity to touch, one patient had overlapping burning pain and numbness, one patient had overlapping electric-like pain and numbness, and one patient had overlapping functional GI with muscle weakness.

^†^One patient had combination symptoms of numbness and functional GI problems.
